# Increased synaptic turnover in injured cortical axons: exploring the role of SARM1 ablation

**DOI:** 10.3389/fnsyn.2026.1741328

**Published:** 2026-02-23

**Authors:** Ensieh Izadi, William Bennett, Jessica Collins, Aidan Bindoff, Anna King, Alison Canty

**Affiliations:** Wicking Dementia Research and Education Centre, University of Tasmania, Hobart, TAS, Australia

**Keywords:** axon injury, axotomy, cortical axons, multiphoton imaging, SARM1, synaptic plasticity

## Abstract

**Introduction:**

Programmed axon degeneration significantly affects neural connectivity, however, the underlying mechanisms remain poorly understood, particularly in cortical regions. Sterile Alpha and TIR motif-containing protein 1 (SARM1) is a known regulator of axon degeneration in the peripheral nervous system, but its role in cortical axon plasticity, particularly during injury conditions, remains unclear. This study examined the role of SARM1 in synaptic connectivity and remodelling in the adult sensory-motor cortex under normal physiological conditions and following acute axonal injury.

**Methods:**

Adult male Thy1-GFP-M mice (3–12 months) expressing EGFP in excitatory neurons were also either wild-type (WT-GFP) or null for SARM1 (SARM1KO-GFP). Using *in vivo* multiphoton microscopy, long cortical axon segments (~335 μm ± 140 μm), with *terminaux* and *en passant* synaptic boutons in the upper layers of the cortical neuropil, were repeatedly imaged at 48-h intervals to assess axon morphology, synaptic density, and synaptic turnover in the presence and absence of SARM1.

**Results:**

Without injury, axon morphology, synaptic density, and turnover were similar between WT and SARM1KO groups, suggesting that SARM1 is not necessary for maintaining baseline cortical synaptic connectivity. Following axotomy by laser lesion, the non-degenerating proximal axon (still connected to the soma) showed significant changes in synaptic plasticity, with an increased rate of loss of synapses.

**Discussion:**

Our findings suggest that SARM1 plays no role in the remodelling of synapses in the proximal axon after an acute axonal injury.

## 1 Introduction

Axon injury significantly impacts neuronal connectivity and compromises the fidelity of synaptic communication within neural circuits. Disrupted neuronal connectivity triggers widespread effects on essential processes, including neurotransmitter homeostasis regulation, calcium signalling, activity-dependent plasticity, and ultimately, reshaping how synapses strengthen or weaken in response to neuronal activity, environmental changes, or injury (Puderbaugh and Emmady, 2025). Along axons, synaptic contacts are organised as dynamic swellings or protrusions, classified as *terminaux* boutons (TB), which exhibit relatively high plasticity, and *en passant* boutons (EPB), which tend to be more stable (Fulopova et al., 2022, 2025). The interplay between synaptic dynamics and stability is thought to underpin circuit adaptability and resilience in the face of trauma, aging and other environmental stressors (Fulopova et al., 2025).

Recent work has converged on Sterile Alpha and TIR domain-containing protein 1 (SARM1) as a central executor of injury-induced axon degeneration (Lin et al., 2014; Maynard et al., 2020; Miyamoto et al., 2024). Upon injury, SARM1 becomes activated, triggering a rapid self-destructive cascade driven by the hydrolysis of NAD+, precipitating metabolic failure and fragmentation of damaged axon segments (Essuman et al., 2017; Gerdts et al., 2015; Osterloh et al., 2012). Although seemingly destructive, selective axon loss can paradoxically facilitate circuit-level plasticity by clearing dysfunctional branches and enabling compensatory remodelling among the remaining surrounding synapses (Gerdts et al., 2016; Shin et al., 2014). Beyond its metabolic role, SARM1 has also been implicated in shaping neuronal structure through effects on cytoskeletal stability and microtubule dynamics, processes central to bouton maintenance, axon branching and ultimately synaptic connectivity (Gerdts et al., 2015; Ketschek et al., 2022). Recent work has shown that SARM1 regulates AMPA receptor endocytosis in hippocampal neurons, suggesting a role in long-term-depression (LTD) and synaptic plasticity (Morishita and Matsuda, 2025).

Despite these advances, key gaps in our understanding remain. Much of our mechanistic understanding derives from peripheral axon degeneration, leaving open questions about SARM1’s role in the adult cerebral cortex. Specifically, it is unclear whether SARM1 contributes to the maintenance of synaptic connectivity in intact adult cortical axons and how it modulates synaptic plasticity within surviving cortical axons after an injury. Clarifying these issues is essential for translating axon protection strategies into therapies to minimise neuronal functional loss while promoting adaptive synaptic remodelling after brain injuries.

Here, we address these questions by combining genetic, optical and microsurgical approaches in adult transgenic mice. The approach builds on recent work that has established robust metrics for synaptic bouton dynamics in the cortex (Doran et al., 2021; Fulopova et al., 2022, 2025; Laperchia et al., 2013). Thy1-GFP-M mice express EGFP in sparse subsets of excitatory cortical axons (Canty et al., 2013b; Feng et al., 2000). Cranial window surgery and *in vivo* multiphoton microscopy allow identification and tracking of individual axons and their synapses over time in the intact brain. We used Thy1-GFP-M transgenic mice as a control group and crossed them with SARM1 knockout mice as the experimental group. We paired longitudinal imaging with laser-mediated axotomy to elicit a precisely timed, spatially restricted axon injury, enabling within-axon comparisons of bouton behaviour before and after injury. We quantified synaptic density (synaptic boutons per unit axon length), synaptic gains, losses and turnover ratio (gains plus losses relative to the total number of boutons) as sensitive readouts of structural plasticity across two types of synaptic boutons (Fulopova et al., 2022, 2025).

We hypothesised that SARM1 deletion would preserve normal baseline synaptic connections in the cortex but alter how synapses remodel after injury by altering axonal energy balance and modifying cytoskeletal stability. By delineating SARM1’s contribution to bouton-level dynamics in intact and injured cortical axons, this study aims to clarify when and how SARM1 should be targeted to optimise synaptic plasticity and functional recovery after traumatic brain injury.

## 2 Methods

### 2.1 Animals

Young adult (3–12 months) male Thy1-GFP-M hemizygotes on a C57BL/6 background (*RRID: IMSR_JAX:007788*) served as the control group (WT-GFP; *n* = 12 mice, 21 axons with 694 boutons, combined axonal length 8.2 mm), and were crossed with SARM1 null mutants (homozygotes) on the same background strain C57BL/6 (*RRID: IMSR_JAX:018069*) for the experimental group (SARM1KO-GFP; *n* = 9 mice; 21 axons with 628 boutons, combined axonal length 6.6 mm). The SARM1 mutant mice used in this study were first reported by Kim et al. (2007), are bred as homozygotes, and no gene product (protein) is detected by Western blot analysis of brain from homozygotes. Genotypes were confirmed during breeding by PCR. All animals were bred at the University of Tasmania’s Cambridge Facility Farm (CFF). Littermates were kept in groups of up to 5 mice per cage, housed under a 12-h light/dark cycle at 22 °C, and had *ad libitum* access to standard chow and water. All experiments involving animals were conducted in compliance with the *University of Tasmania Animal Ethics Committee* guidelines and approved under the *Australian Code for the Care and Use of Animals for Scientific Purposes*, 8th edition (National Health and Medical Research Council, 2013).

### 2.2 Cranial window surgery

A modified protocol (Holtmaat and Svoboda, 2009) was used. Adult mice received a subcutaneous injection of analgesia (buprenorphine 0.1 mg/kg) at least 30 min before surgery. Anaesthesia was induced with 5% isoflurane, then maintained at 1.8–2.5% isoflurane in 100% oxygen flow at 0.6–1.0 L/min. Once unconscious, animals were secured in a stereotaxic frame on a heat pad, and the scalp was infiltrated with a local anaesthetic (Bupivacaine 0.25%) before incision. The cranial window was positioned over the primary somatosensory cortex (S1) on the right hemisphere, with the centre approximately 1.5 mm posterior to bregma and 2.5 mm lateral to the midline. This region was chosen due to its accessibility and suitability for imaging, consistent with earlier studies on synaptic dynamics in cortical axons (Canty et al., 2020; Canty et al., 2013a,b). A micro drill with a 0.5 mm burr was used to remove a 3 mm diameter piece of calvarial bone. Sterile artificial cerebrospinal fluid (aCSF) buffer (125 mM NaCl, 5 mM KCl, 10 mM glucose, 10 mM HEPES, 2 mM CaCl2, 2 mM MgSO_4_, pH 7.3–7.4; ~300 mOsm) was applied regularly to cool the surface and remove any bone dust during drilling. The surface of the intact dura was irrigated with sterile gel foam (Pfizer) soaked in artificial cerebrospinal fluid (aCSF), and dexamethasone (4 mg/mL) was applied to reduce inflammation, improve clarity, and maintain proper moisture levels (Park et al., 2015). The craniotomy was covered with a round glass coverslip (5 mm in diameter) and then sealed using Loctite 454 glue and Heraeus Paladur dental cement (Canty et al., 2020; Tang et al., 2021). A titanium bar (10 mm, M2 bolt hole) was attached for imaging stability. Mice recovered for approximately 2–3 weeks before undergoing imaging.

### 2.3 *In vivo* two-photon microscopy

*In vivo* two-photon imaging was performed using a custom-built laser-scanning microscope (Scientifica) equipped with galvo-galvo mirrors, an Olympus 20X water-immersion objective (NA = 1.0, Zeiss Plan-Apo), and a high-sensitivity GaAsP non-descanned photomultiplier detector (Hamamatsu). Femtosecond pulsed infrared excitation was delivered by a mode-locked Ti-sapphire laser (Mai Tai, DeepSee, Spectra-Physics) tuned to 910 nm with group velocity dispersion compensation. Laser power delivered to the back aperture ranged from 20 to 90 mW, depending on the imaging depth, which enabled imaging deeper than 100 μm into the cortex while minimising bleaching of fluorophores or thermal damage. Anaesthesia induction for imaging was initiated and maintained as described earlier. To maintain stability and reduce motion artifacts, the titanium head bar was secured to the stereotaxic stage mounted on an air table to minimise vibrations and ensure stability. The vascular landmark correlation in the window was used to identify the same region in subsequent imaging sessions.

After switching from bright-field to two-photon mode, the labelled neuropil appeared as a dense meshwork of GFP-labelled axons and dendrites. All images for analysis were captured as image stacks (512 × 512 pixels per slice with an XY resolution of 0.2 μm per pixel and a Z-step of 2 μm between slices) using ScanImage 3.8.1 software (Vidrio Technologies, LLC; Pologruto et al., 2003) incorporating the Navigator plugin (Meyers, 2013) in MATLAB. The same images were captured across repeated sessions.

### 2.4 Axon selection

During the first imaging session for each mouse, the GFP-labelled neuropil across the upper layers of the sensory-motor cortex was searched to an average depth of 100 μm, to identify clear axons for imaging. Axons were selected based on three main characteristics: defined terminal endings, number of synapses, and length (at least 150 μm, minimum 14 boutons). Each visible synapse was identified and monitored at 48-h intervals to assess its stability. Since terminal axons of sufficient length were difficult to locate, axons that extended beyond the imaging window (i.e., without visible endings) were also included, to increase sampling and avoid bias toward only short or truncated axons. At the point of injury, care was taken to ensure that the surviving segments of the axon (connected to the cell body) contained at least 14 synapses after the injury (post-axotomy).

**Figure 1:**
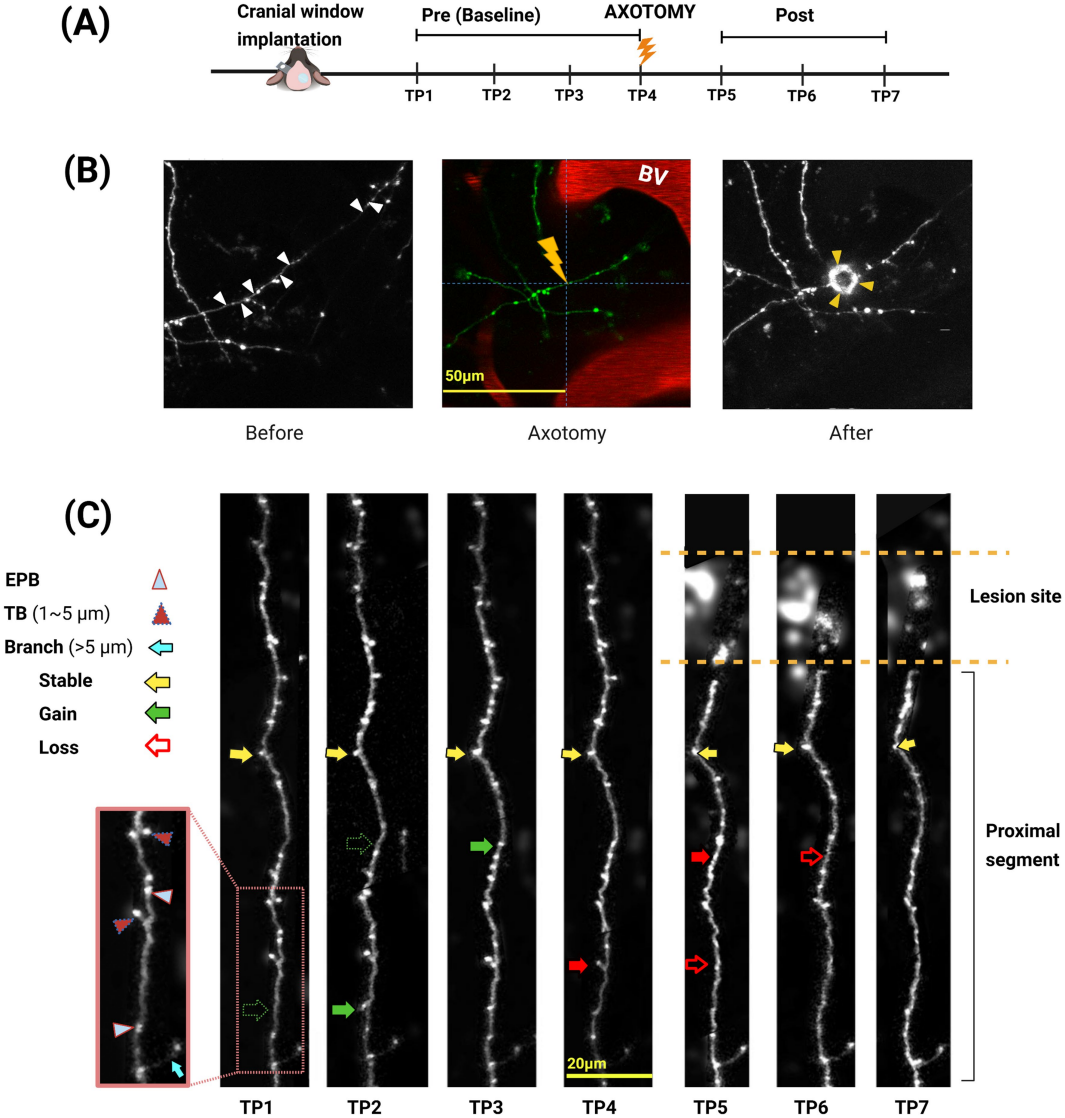
*In vivo* two-photon imaging of cortical axons and synaptic dynamics following laser-induced axotomy. **(A)** Schematic of the experimental timeline. Baseline imaging was performed at TP1 (−6 days), TP2 (−4 days), TP3 (−2 days), TP4 (day 0), with axotomy immediately after TP4, and post-axotomy TP5 (+2 days), TP6 (+4 days), and TP7 (+6 days). **(B)** Representative example of targeted axotomy. Left panel shows target axon (white arrowheads). Middle panel shows dual labelling with blood vessels in red and identified lesion site. Right panel shows lesioned axon 2 min after injury. A successful axotomy appears as a hollow ring at the lesion site, indicated by yellow arrowheads. **(C)** Longitudinal high-resolution imaging of synaptic bouton dynamics. Boutons were classified as *en passant* boutons (EPB; light blue arrowheads), *terminaux* boutons (TB, 1–5 μm; red arrowheads), or branches (>5 μm; cyan arrow). Stable boutons are denoted with yellow arrows, gained boutons with filled green arrows, and lost boutons with red arrows. Open red arrows indicate boutons that were lost and confirmed absent at subsequent timepoints within 48-h intervals. Lightning bolt indicates axotomy.

Images were captured at the same time of day, 48 h apart, across seven imaging sessions, with a targeted laser lesion applied immediately after image collection in the 4th session. The baseline and post-axotomy imaging sessions are as follows: timepoints 1, 2, 3, and 4 (TP1 to TP4) prior to the lesion (baseline) and post-axotomy timepoints 5, 6, and 7 (TP5 to TP7, Figure 1A).

### 2.5 Axotomy

A red fluorescent dye (1 mM Sulforhodamine-B, 10 mL/kg i.p.) was administered at the start of imaging session 4 to visualize a vascular map of the brain (Kovalchuk et al., 2015), which assisted in targeting axotomy sites away from blood vessels that could rupture. The laser was tuned to 850 nm to image this vascular marker simultaneously with GFP.

**Figure 2:**
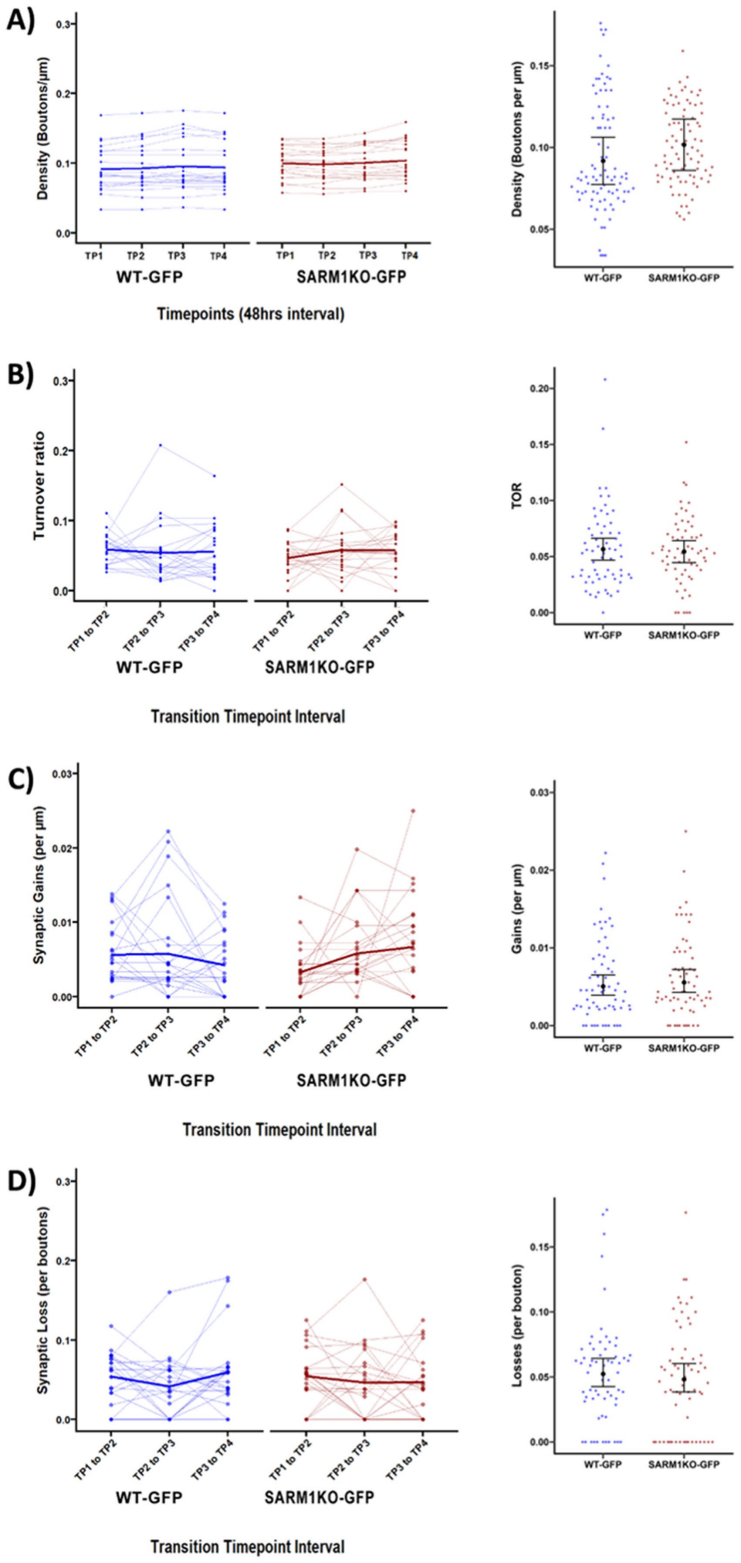
Baseline synaptic dynamics of WT-GFP and SARM1KO-GFP cortical axons. For all panels, the within axon trajectories over time are shown on the left, and on the right, is the aggregated pre- and post-axotomy data with estimated marginal means and 95% CI over beeswarm plots of raw observations. **(A)** The synaptic density was stable for WT-GFP (blue, left) and SARM1KO-GFP (red, right) axons (thin lines) over 48-h time intervals, with the mean indicated by a thicker line. The EMMs and 95% confidence intervals for synaptic density at each interval (illustrated in the dot plot of synaptic density for each axon at each time point) showed no statistically significant interaction between genotype and timepoints, indicating stable density across the baseline period in both genotypes (right panel). **(B)** TOR of individual axons across three 48-h intervals (TP1–TP2, TP2–TP3, TP3–TP4) was comparable between genotypes. The EMMs and 95% confidence intervals for TOR at each interval (illustrated in the dot plot of TOR for each axon at each 48-h interval) showed no statistically significant interaction between genotype and timepoints, indicating stable TOR values across the baseline period in both genotypes. **(C,D)** Synaptic gains **(C)** and losses **(D)** at each 48-h interval showed no significant differences, except for the interaction between gains per μm from TP3 to TP4; in SARM1KO-GFP, gains per μm increased significantly (*p* < 0.001). *n* = 21 axons per genotype. Across all panels, WT-GFP (blue, left) and SARM1KO-GFP (red, right).

A complete set of images was collected before axotomy to establish a baseline for comparison. The microlesion (at least 25 μm away from blood vessels) was created by setting the laser to a wavelength of 850 nm to deliver a pulse at 100% laser power with a peak power of 1.2–1.4 W. A “scan” of 32 × 32 pixels was performed without XY movement, ensuring that the laser was focused on a central spot of approximately 3 μm diameter. The frame rate was 5.92 Hz, so capturing one frame meant the shutter remained open for approximately 170 ms. An estimated 50% of delivered power was lost in the objective and glass (Holtmaat and Svoboda, 2009), so only approximately 0.7 W of laser power (1.4 W max) reached the cortex, and a total energy of 120 mJ (0.7 W × 170 ms) was calculated to be delivered to the lesion site. An energy density of approximately 1.68 MJ/cm^2^ was applied to the 3 μm circular lesion area, effectively severing the axon while minimising damage to adjacent tissues. Lesions were deemed successful when a clear cut in the axon shaft was visible, often (but not always) accompanied by a transient bright “halo” around the lesion site (Figure 2B). We often observed limited, temporary dysmorphology in nearby axons, which typically resolved within 20 to 30 min.

### 2.6 Synapse identification

All image stacks were processed using ImageJ software (Fiji, 2.1.0, Schindelin et al., 2012) starting with 16-bit (intensity value 0–65,535) greyscale images from the microscope. The process typically involved structuring raw data, converting images into an 8-bit format (with intensity values ranging from 0 to 255), validating their quality, reducing noise, and normalising them. Maximum projections were used to montage overlapping frames together and verify axon length and morphology. Curation ensured the images were formatted, montaged, and reliable, facilitating subsequent analytical stages. Adobe Photoshop CS2 and XuvStitch 1.8.099 (Xuvtools license 1.0, Emmenlauer et al., 2009) were used to montage maximum projections and z-stacks, respectively. All the images and montages were saved in Tagged Image File Format (TIFF) (Linkert et al., 2010).

Synaptic boutons were classified as *terminaux* boutons (TB) or *en passant* boutons (EPB) following De Paola et al. (2006). Unlike earlier reports that described exclusively TB-rich or EPB-rich populations, most cortical axons in the Thy1-GFP-M mice used in this study exhibited a mixture of TBs and EPBs. The literature indicates that TBs exhibit greater plasticity compared to EPBs (Canty et al., 2013a,b; De Paola et al., 2006; Fulopova et al., 2025), and the relative abundance of each type was quantified for each axon, ranging from 10 to 75% TBs. Within each axon, these percentages were averaged across imaging sessions.

### 2.7 Quantification of synaptic dynamics

Synaptic boutons were annotated using custom MATLAB scripts included with ScanImage (scim_spineAnalysis.m) (Holtmaat and Svoboda, 2009). Each bouton was assigned a unique identifier to track its status (stable, lost, or gained) across consecutive imaging sessions. Axon segment length was measured to normalise bouton counts and account for length variability, with the same segment consistently analysed at each timepoint.

Bouton classification followed established morphological criteria. EPBs were defined as a swelling visible in at least two optical slices and at least twice the GFP labelling signal of the surrounding axon shaft (Figure 1C, light blue arrowheads). TBs were identified as protrusions 1–5 μm in length, present in at least two consecutive z-slices (2 μm apart; Figure 1C, dark red arrowheads). We marked EPBs as either present or absent by manual annotation, and TBs were measured by drawing a line along their length. Structures exceeding 5 μm were classified as short branches (Figure 1C, blue arrow) and excluded from the analysis. Identification of EPB and TB synapses in Thy1-GFP mice is a commonly used approach for investigating synaptic dynamics in the intact brain, with (i) both gains and losses of boutons over time and (ii) electron microscopy reconstruction demonstrating active zones at GFP swellings along the axon or in terminal boutons confirmed in previous studies by this authorship team, and others (Bass et al., 2025; Bass et al., 2017; De Paola et al., 2006; Canty et al., 2013a; Fulopova et al., 2022, 2025; Holtmaat et al., 2008).

Data were exported to R for analysis using a custom script. Synaptic density was calculated as the number of boutons per unit length of axon. Synaptic turnover ratio (TOR) between two consecutive time points (A to B) was calculated as:


$$\mathrm{TOR}=\frac{{\text{Gains}}_{B}+{\text{Losses}}_{B}\;}{\;{\text{Bouton}}_{A}+{\text{Bouton}}_{B}}$$

We applied a minimal threshold of 0.1 μm to register bouton presence at any length, annotating and correlating across time (gain, loss, and stable). EPBs were classified as present or absent, while TBs had to reach at least 1.0 μm in one session to be included. The final dataset included a unique mouse ID, axon segment number, axon length, number of boutons (including EPB and TB), TOR, and gains and losses at each time point. TB% was calculated as:


$$\mathrm{TB}\%=\frac{\mathrm{TB}\;}{\left(\mathrm{EPB}+\mathrm{TB}\right)}\times 100$$

### 2.8 Statistical approach

Statistical analyses and visualisations were performed in R4.1.0 (R Core Team, 2021) using generalised linear mixed models (GLMMs) fitted with *lme4* (v1.1–37; Bates et al., 2015) and the *glmmTMB* (v1.1.11; Brooks et al., 2017) packages. In contrast, GLMMs were employed to accommodate a wide range of statistical distributions under a hierarchical design. We report *p*-values (<0.05 as statistically significant) alongside effect sizes (standardised *β* coefficients) to convey the magnitude of effects. For linear mixed-effects models (LMMs) or GLMMs, marginal and conditional *R*^2^ quantified variance explained by fixed effects alone is calculated to assess model fit and effect size. Marginal *R*^2^ represents the proportion of variance explained by the fixed effects alone, versus fixed plus random effects (Nakagawa and Schielzeth, 2013), aiding interpretation in the presence of biological variability (Nebe et al., 2023). To account for the hierarchical design (multiple axons per mouse), all mixed models included random intercepts for unique mouse ID with axons nested within ID: (1| ID/axon). Fixed effects were genotypes (WT-GFP, SARM1KO-GFP), timepoint (48-h intervals), TB%, and the genotype × timepoint interactions.

*Model 1: Synaptic density and turnover ratio (Gaussian LMM):*


$$\begin{array}{l} \text{genotype}+\text{timepoints}+\mathrm{TB}\%+(\text{genotype}\times \text{timepoints})+\\ \left(1|\mathrm{ID}/\text{axon}\right) \end{array}$$

*Model 2: Synaptic gains (negative binomial GLMM with length offset):*


$$\begin{array}{l} \;\text{genotype}+\text{timepoint}+\mathrm{TB}\%+(\text{genotype}\times \text{timepoints})+\\ \text{offset}\;(\log\;(\text{Length}))+(1|\mathrm{ID}/\text{axon}) \end{array}$$

An offset term of log (Length) was included to normalise synaptic gains by segment size, reflecting that longer axons can host more boutons. Treating length as exposure allows fair composition of gain rates across genotypes and timepoints, independent of segment size. Thus, the model estimates a bouton gain rate per length rather than raw counts.

*Model 3: Synaptic losses (poisson GLMM with prior- bouton offset):*


$$\begin{array}{l} \;\text{genotype}+\text{timepoint}+\mathrm{TB}\%+(\text{genotype}\times \text{timepoints})+\\ \text{offset}(\log(\mathrm{pre}.\mathrm{no}.\text{Boutons}))+(1|\mathrm{ID}/\text{axon}) \end{array}$$

This formula explained the variability in synaptic loss (response variable) by reflecting the effects of SARM1 removal and TB percentage at each timepoint, along with previous values of the total number of boutons, and by accounting for axons nested within mouse ID as random effects.

## 3 Results

### 3.1 Baseline circuit morphology and synaptic density are stable and comparable in WT-GFP and SARM1KO-GFP axons

#### 3.1.1 Morphology

To examine baseline cortical axon structure, we identified a total of 21 axons from 12 WT-GFP mice. Depending on the density of labelling and the window clarity, between 1 and 4 cortical axons were identified in each brain, containing a mixture of TBs and EPBs, with an average length of 370 μm (±160 μm) and containing 35 (±16) boutons. Axons were thin projections with a uniform diameter and width, stretching across cortical layers and descending into deeper structures and contained a mixture of synaptic boutons, either TBs or EPBs. Axon morphology varied across the imaged region and field of view, with axons ranging from unbranched to highly branched. Bouton presence varied independently of branching, although synaptic-rich axons were more often branched.

In SARM1KO mice, we used the same criteria to investigate potential differences in morphology and distribution compared to the WT-GFP counterparts. A comparable dataset of 21 axons was obtained from 9 SARM1KO-GFP mice, with between 1 and 4 axons per mouse, an average length of 370 μm (±160), and 31 (±11) boutons. Cortical axons in the SARM1KO-GFP brain showed a similar synapse distribution along the thin axonal backbone. Morphology, bouton distribution, and branching were indistinguishable from those of WT-GFP, suggesting that SARM1 deletion does not alter the baseline structural aspects of cortical axons.

Mean TB% was 27.5% for WT-GFP and 33.0% for SARM1KO-GFP. The estimated difference between genotypes was 5.53 percentage points, with a 95% CI of −4.18 to + 15.24, which was not statistically significant. As the data were not normally distributed, a Wilcoxon rank-sum test was used: *W* = 180, *p* = 0.314.

#### 3.1.2 Synaptic density

Baseline synaptic density was quantified across TP1-TP4 (pre-axotomy) using a linear mixed-effects model fitted with random intercepts for axons nested within mice to assess the effects of genotype, timepoints, and TB% on synaptic density in WT-GFP and SARM1KO-GFP axons. Group means of synaptic density between genotypes were 0.094 boutons/μm for WT-GFP and 0.10 boutons/μm for SARM1KO-GFP, with no genotype effect [*β* = 3.8 × $10^{-3}$ , 95% CI (−0.01, 0.02) *p* = 0.678; Figure 2A]. Table 1 shows the “omnibus” statistic for all models, with the chi-squared test indicating which predictors contributed significantly to the model. Density remained stable for both genotypes across timepoints, with only a modest, genotype-independent increase detected at TP3 relative to TP1 (*β* = 4.3× $10^{-3}$ , *p* = 0.016; standardised *β* = 0.14), explaining minimal variance (Table 2).

**Table 1:** Omnibus statistics for overall models.

Predictor	Density	TOR	Gains	Losses
Baseline				
Genotype	Chi-squared = 0.763 *p* = 0.382	Chi-squared = 0.047 *p* = 0.829	Chi-squared = 0.007 *p* = 0.936	Chi-squared = 0.104 *p* = 0.748
Time	Chi-squared = 1.021 *p* = 0.796	Chi-squared = 0.444 *p* = 0.801	Chi-squared = 1.722 *p* = 0.423	Chi-squared = 1.889 *p* = 0.389
TB%	Chi-squared = 8.933 *p* = 0.003	Chi-squared = 0.205 *p* = 0.651	Chi-squared = 6.395 *p* = 0.01	Chi-squared = 2.012 *p* = 0.156
Genotype*Time	Chi-squared = 0.495 *p* = 0.920	chi-squared = 1.975 *p* = 0.373	Chi-squared = 0.22 *p* = 0.016	Chi-squared = 1.012 *p* = 0.603
Post-axotomy				
Genotype	Chi-squared = 0.788 *p* = 0.375	Chi-squared = 0.9465 *p* = 0.331	Chi-squared = 0.688 *p* = 0.407	Chi-squared = 0.04 *p* = 0.841
Time	Chi-squared = 9.06 *p* = 0.17	Chi-squared = 18.29 *p* = 0.003	Chi-squared = 6.40 *p* = 0.269	Chi-squared = 14.77 *p* = 0.011
TB%	Chi-squared = 4.147 *p* = 0.042	Chi-squared = 0.418 *p* = 0.518	Chi-squared = 1.02 *p* = 0.313	Chi-squared = 0.376 *p* = 0.54
Genotype*Time	Chi-squared = 4.784 *p* = 0.572	Chi-squared = 6.358 *p* = 0.273	Chi-squared = 3.359 *p* = 0.645	Chi-squared = 5.79 *p* = 0.327

**Table 2:** Summary of statistical analysis of baseline synaptic density, turnover ratio (TOR), gains and losses across genotypes, timepoints, and terminal bouton percentage (TB%).

Predictor		Density (boutons/μm)	TOR		Gain (per μm)	Loss (per bouton)
Genotypes		*β*: 0.04 (std. 0.13), 95%CI [−0.01, 0.02] [std. (−0.48, 0.74)], *p*: 0.68	*β*: −0.01 (std. −0.35), 95%CI [−0.06, 0.02] [std. (−0.98, 0.28)], *p*: 0.27		*β*: −0.54 (std. −1.0), 95%CI [−1.1, 0.007] [std. (−2.3, 0.25)], *p*: 0.053	*β*: 0.01 (std. −1.0), 95%CI [−0.5, 0.5] [std. (−1.1, 1.1)], *p*: 0.97
Timepoint	2	*β*: 0.001 (std. 0.04), 95%CI [−0.0022, 0.0048] [std. (−0.07, 0.16)], *p*: 0.47	2–3	*β*: −0.05 (std. −0.15), 95%CI [−0.02, 0.01] [std. (−0.69, 0.40)], *p*: 0.60	*β*: 0.02 (std. 0.0000002), 95%CI [−0.39, 0.43] [std. (−0.52, 0.52)], *p*: 0.92	*β*: −0.26 (std. −0.4), 95%CI [−0.73, 0.21] [std. (−1.1, 0.3)], *p*: 0.28
	3	*β*: 0.004 (std. 0.14), 95%CI [0.00082, 0.00775] [std. (0.03, 0.26)], *p*: 0.016	3–4	*β*: −0.03 (std. −0.09), 95%CI [−0.02, 0.01] [std. (−0.64, 0.45)], *p*: 0.73	*β*: −0.28 (std. -0.11), 95%CI [−0.73, 0.17] [std. (−0.65, 0.43)], *p*: 0.22	*β*: 0.09 (std. −0.01), 95%CI [−0.33, 0.52] [std. (−0.59, 0.56)], *p*: 0.67
	4	*β*: 0.003 (std. 0.09), 95%CI [−0.00094, 0.00599] [std. (−0.03, 0.20)], *p*: 0.153				
TB %		*β*: 0.0009 (std. 0.46), 95%CI [0.02, 0.79] [std. (0.19, 0.74)], *p*: 0.001	*β*: −0.0001 (std. −0.05), 95%CI [−0.0006, 0.0004] [std. (−0.27, 0.17)], *p*: 0.66		*β*: 0.014 (std. 0.52), 95%CI [0.003, 0.02] [std. (−0.71, 1.75)], *p*: 0.011	*β*: −0.007 (std. −0.005), 95%CI [−0.02, 0.003] [std. (−0.42, 0.40)], *p*: 0.16
Genotype and timepoints interactions	2	*β*: −0.003 (std. -0.10), 95%CI [−0.008, 0.06] [std. (−0.29, 0.04)], *p*: 0.22	2–3	*β*: 0.02 (std. 0.49), 95%CI [−0.009, 0.04] [std. (−0.28, 1.3)], *p*: 0.21	*β*: 0.56 (std. 0.51), 95%CI [−0.1, 1.2] [std. (−0.63, 1.65)], *p*: 0.096	*β*: 0.09 (std. 0.21), 95%CI [−0.6, 0.8] [std. (−0.75, 1.16)], *p*: 0.79
	3	*β*: −0.004 (std. −0.13), 95%CI [−0.009, 0.0012] [std. (−0.29, 0.04)], *p*: 0.14	3–4	*β*: 0.01 (std. 0.42), 95%CI [−0.01, 0.04] [std. (−0.35, 1.2)], *p*: 0.28	*β*: 0.99 (std. 1.2), 95%CI [0.3, 1.8] [std. (0.5, 1.8)], *p*: 0.004	*β*: −0.24 (std. −0.15), 95%CI [−0.9, 0.4] [std. (−1.0, 0.72)], *p*: 0.46
	4	*β*: 0.001 (std. 0.04), 95%CI [0.004, 0.006] [std. (−0.13, 0.21)], *p*: 0.63				

Furthermore, Estimated Marginal Means (EMMs) averaged over model variables, including timepoint, TB%, axon number, and mouse ID, confirmed the absence of a significant genotype effect. The model explained 96.4% of the total variance (conditional *R*^2^ = 0.964) with the fixed effects (genotype, timepoints, TB and their interaction) contributing for 22.1% (marginal *R*^2^ =0.221). Together, these results demonstrate that baseline cortical axon morphology and synaptic density remain stable across time and are comparable between WT-GFP and SARM1KO-GFP mice.

### 3.2 Baseline synaptic turnover ratio was similar across groups, but SARM1 deletion alters remodelling dynamics by enhancing bouton formation while leaving bouton elimination unaffected

#### 3.2.1 Synaptic turnover

We evaluated bouton remodelling under baseline conditions by measuring the synaptic turnover ratio (TOR) across TP1-TP4 to determine whether SARM1 deletion affects synaptic formation or elimination. Mixed-effects modelling confirmed that there were no main effects of genotype, time intervals, or their interaction. Thus, the synaptic turnover ratio appeared to be unaffected by SARM1 ablation. On average, TOR was comparable between groups (WT-GFP: 0.057 ± 0.036; SARM1KO-GFP: 0.054 ± 0.030, *p* = 0.72, Figure 2B), indicating no significant difference in overall synaptic turnover. When assessing changes in TOR across time, we found no significant trend (Table 2), suggesting that TOR remained stable across the imaging intervals.

#### 3.2.2 Synaptic gains

Using mixed-effects modelling, we found that SARM1KO-GFP axons began with fewer synaptic bouton gains per unit axonal segment length, compared to WT-GFP (*β* = −0.54, *p* = 0.052, Figure 2C). However, SARM1KO-GFP axons subsequently showed an increase in bouton formation by the next imaging session, ultimately surpassing WT-GFP between transition timepoints 3 and 4 (*β* = 0.99, *p* = 0.004). This may suggest a modest increase in gains; however, it is unlikely to be biologically or functionally significant under baseline conditions with stable density and TOR.

#### 3.2.3 Synaptic losses

Both WT-GFP and SARM1KO-GFP axons showed fluctuations in synaptic loss at the level of individual axons, but no consistent group-level differences were detected (Figure 2D). Loss rates did not differ between genotypes (*β* = 0.01, *p* = 0.97) and remained stable across time (TP2-TP3: *β* = −0.26, *p* = 0.28; TP3-TP4: *β* = 0.09, *p* = 0.67), with no significant interaction between genotype across time (all *p* > 0.05). Although losses varied across axons, overall bouton elimination remained stable during baseline imaging in both groups.

In summary, the cortical axons included in this study from WT-GFP and SARM1KO-GFP mice can be considered comparable in terms of morphology, synapse types and dynamics (TB%, density, TOR, gains, losses). Among density, TOR, loss, and gain, only gain showed a significant effect over time in SARM1KO-GFP (*p*-value = 0.004). The consistency of density, TOR, and loss indicates that difference in genotype does not alter synaptic density or turnover across 48 h imaging sessions.

### 3.3 Laser-mediated axotomy

Following axotomy, the disconnected distal segment from the cell body typically exhibited morphological change after a delay of minutes to hours, followed by rapid degeneration. The surviving proximal stump exhibited an immediate injury response characterised by the rapid formation of axonal varicosities (“beading”) and focal swelling along the axon shaft that resolved within 20–30 min, similar to prior reports (Beirowski et al., 2010; Canty et al., 2013b; Gu et al., 2017). No regrowth of the severed stump was observed in either genotype for at least 14 days post-injury.

### 3.4 Synaptic density remains stable after axotomy, independent of genotype

**Figure 3:**
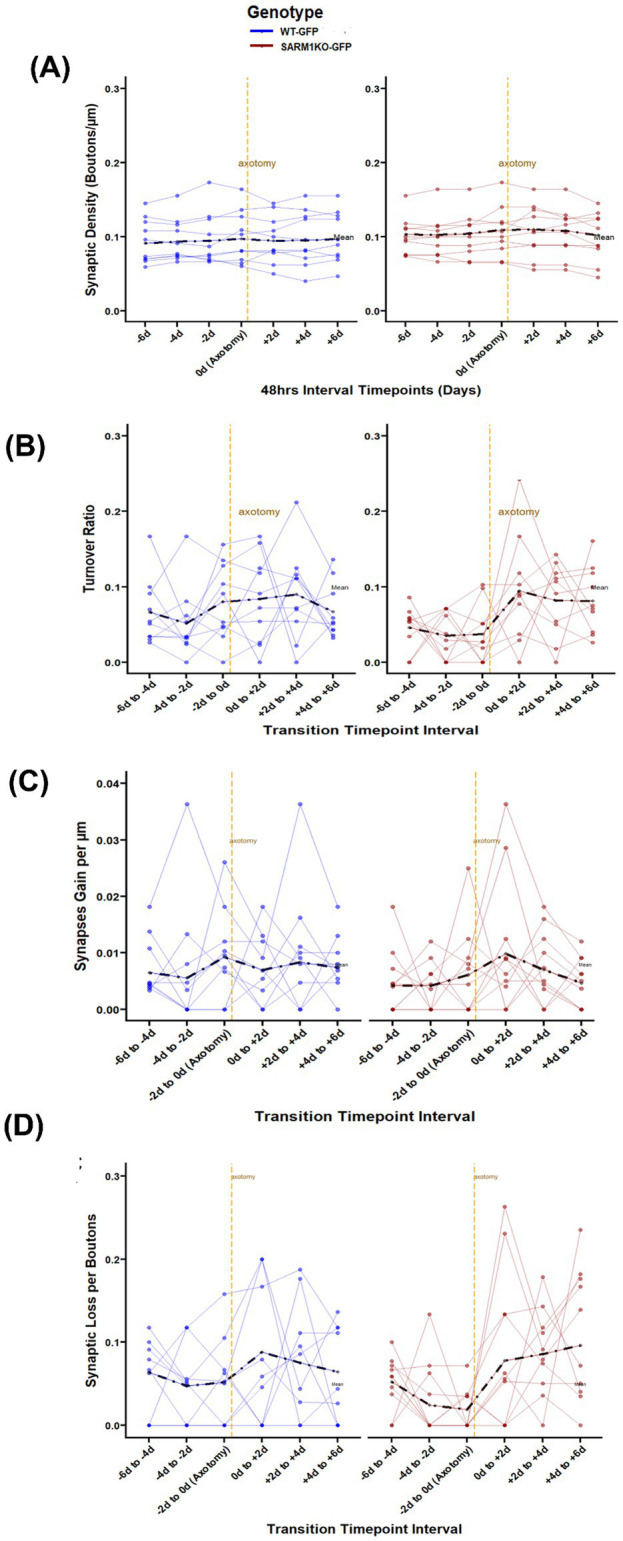
Synaptic remodelling pre- and post-axotomy in adult cortical axons in WT-GFP and SARM1KO-GFP mice. **(A)** Synaptic density remained stable post-axotomy for both WT-GFP and SARM1KO-GFP mice. **(B)** The turnover ratio post-axotomy was comparable in both genotypes. **(C)** After axotomy, the rates of WT-GFP and SARM1KO-GFP gains remained stable. **(D)** Both WT-GFP and SARM1KO-GFP axons showed increased synaptic loss immediately following axotomy. Across all panels, WT-GFP (blue, left) and SARM1KO-GFP (red, right). Thick dotted lines indicate mean. Orange dotted line indicates axotomy.

A total of 20 axons (WT-GFP = 10 axons in 5 mice, and SARM1KO-GFP = 10 axons in 7 mice) were imaged at 48 intervals before and after axotomy, a subset of the axons previously discussed (see Figure 2). Initially, a Welch *t*-test was performed on the data distribution to compare synaptic density pre-axotomy as well as post-axotomy. This step was essential to show that there were no significant differences between the two genotypes (all *p* > 0.05), confirming comparable starting conditions for the proximal segments. Once this equivalence was established, the post-axotomy analysis was conducted for the same axons at TP5, TP6 and TP7. We used a similar linear mixed-effects model approach as for baseline data, with the addition of a fixed effect for timepoint relative to injury. We found no significant differences in density post-axotomy (Table 3, Figure 3A).

**Table 3:** Summary of statistical analysis of synaptic density, turnover ratio (TOR), gains and losses across genotypes, time points, and terminal bouton percentage (TB%) pre- and post-axotomy.

Predictor		Density (boutons/μm)	TOR		Gain (per μm)	Loss (per bouton)
Genotypes		*β*: 0.01 (std. 0.38), 95%CI [−0.01, 0.04] [std. (−0.4, 1.2)], *p*: 0.35	*β*: −0.02 (std. −0.35), 95%CI [−0.06, 0.02] [std. (−1.2, 0.4)], *p*: 0.3		*β*: −0.43 (std. −0.2), 95%CI [−1.3, 0.5] [std. (−0.2, 1.6)], *p*: 0.34	*β*: −0.19 (std. −0.9), 95%CI [−1.0, 0.66] [std. (−2.8, 1.0)], *p*: 0.66
Timepoint/time intervals	5	*β*: 0.003 (std. 0.10), 95%CI [−0.004, 0.01] [std. (−0.1, 0.3)], *p*: 0.4	4–5	*β*: 0.02 (std. 0.4), 95%CI [−0.02, 0.05] [std. (−0.4, 1.1)], *p*: 0.35	*β*: 0.07 (std. 0.4), 95%CI [−0.66, 0.80] [std. (−0.4, 1.2)], *p*: 0.85	*β*: 0.35 (std. −0.12), 95%CI [−0.39, 1.08] [std. (−1.1, 0.9)], *p*: 0.35
	6	*β*: 0.004 (std. 0.13), 95%CI [0.003, 0.01] [std. (−0.1, 0.4)], *p*: 0.27	5–6	*β*: 0.02 (std. 0.5), 95%CI [−0.01, 0.06] [std. (−0.3, 1.2)], *p*: 0.21	*β*: 0.25 (std. −0.11), 95%CI [−0.45, 0.95] [std. (−0.8, 1.01)], *p*: 0.48	*β*: 0.15 (std. −0.27), 95%CI [−0.61, 0.91] [std. (−1.3, 0.8)], *p*: 0.70
	7	*β*: 0.006 (std. 0.18), 95%CI [−0.0015, 0.01] [std. (−0.05, 0.40)], *p*: 0.13	6–7	*β*: 0.0005 (std. 0.01), 95%CI [−0.04, 0.04] [std. (−0.7, 0.8)], *p*: 0.98	*β*: 0.13 (std. 0.3), 95%CI [−0.58, 0.85] [std. (−0.6, 1.2)], *p*: 0.72	*β*: 0.008 (std. −0.01), 95%CI [−0.78, 0.79] [std. (−1.2, 0.99)], *p*: 0.98
TB %		*β*: 0.0009 (std. 0.41), 95%CI [0.00003, 0.002] [std. (0.02, 0.79)], *p*: 0.042	*β*: −0.0003 (std. −0.07), 95%CI [−0.001, 0.0005] [std. (−0.3, 0.1)], *p*: 0.52		*β*: 0.008 (std. −0.1), 95%CI [0.003, 0.02] [std. (−0.78, 0.6)], *p*: 0.33	*β*: −0.006 (std. −0.4), 95%CI [−0.02, 0.008] [std. (−0.90, 0.08)], *p*: 0.38
Genotype and timepoints interactions	5	*β*: 0.003 (std. 0.11), 95%CI [−0.007, 0.01] [std. (−0.21, 0.43)], *p*: 0.5	4– 5	*β*: 0.03 (std. 0.6), 95%CI [−0.02, 0.08] [std. (−0.43, 1.7)], *p*: 0.25	*β*: 0.78 (std. 0.6), 95%CI [−0.3, 1.9] [std. (−0.63, 1.65)], *p*: 0.6	*β*: 0.03 (std. −1.0), 95%CI [−1.0, 1.1] [std. (−2.7, 0.6)], *p*: 0.96
	6	*β*: 0.0008 (std. 0.03), 95%CI [−0.009, 0.01] [std. (−0.29, 0.34)], *p*: 0.87	5– 6	*β*: 0.01 (std. 0.24), 95%CI [−0.04, 0.06] [std. (−0.8, 1.3)], *p*: 0.65	*β*: 0.26 (std. 1.2), 95%CI [−0.3, 1.3] [std. (0.5, 1.8)], *p*: 0.64	*β*: 0.35 (std. 0.23), 95%CI [−0.7, 1.4] [std. (−1.3, 1.72)], *p*: 0.52
	7	*β*: −0.007 (std. −0.2), 95%CI [−0.02, 0.003] [std. (−0.5, 0.1)], *p*: 0.2	6– 7	*β*: 0.03 (std. 0.7), 95%CI [−0.02, 0.09] [std. (−0.4, 1.8)], *p*: 0.2	*β*: 0.26 (std. 1.2), 95%CI [−1.2, 1.1] [std. (−1.6, 1.5)], *p*: 0.96	*β*: 0.61 (std. 1.0), 95%CI [−0.5, 1.7] [std. (−0.54, 2.6)], *p*: 0.52

### 3.5 Turnover increases post-axotomy, but SARM1 deletion does not alter this

Mixed-effect modelling showed a significant increase in TOR over time in the post-axotomy proximal axon (timepoint as a main effect over the entire model, *p* < 0.002). Neither the main effect of genotype, nor the interaction between time and genotype was significant, suggesting that the pattern of TOR change over time did not differ significantly between the WT-GFP and SARM1KO-GFP groups (Figure 3B). No individual time interval or interaction term reached significance in the model (all *p* > 0.05), and TB% had no detectable influence on TOR (*p* = 0.52). The stable synaptic density, coupled with modest increases in turnover, is consistent with the typical pattern observed in the control group in which SARM1 is present, aligning with previously reported studies (Bradshaw et al., 2021; Canty et al., 2013b; Chen et al., 2021).

### 3.6 Post-axotomy synaptic gains are unaltered and synaptic losses increase with time

Synaptic remodelling following axotomy was assessed by separately analysing bouton formation and elimination using mixed-effects models. For bouton formation (gains), no significant main effect of time or genotype was observed (Table 2; Figure 3C). In contrast, synaptic losses showed a significant main effect of timepoint (*p* = 0.016), suggesting time-dependent modulation of synaptic loss (Figure 3D). However, neither the main effect of genotype [*β* = −0.19, 95% CI (−1.04, 0.66), *p* = 0.660] nor the genotype × time interaction was significant. No individual time interval or interaction term were significant in the model (all *p* > 0.05),

Together, these findings indicate that synaptic density is maintained after axotomy in both WT-GFP and SARM1KO-GFP axons. There is an increase in synaptic loss rates, but this is not enough to lower overall density. SARM1 deletion does not alter synaptic density, TOR, losses or gains post-axotomy. TB% does not significantly alter any of these parameters.

## 4 Discussion

### 4.1 SARM1 deletion does not affect synaptic dynamics in uninjured adult cortical axons

Two-photon imaging revealed that baseline cortical axon morphology is comparable in Thy1-GFP wild-type and SARM1KO-GFP cortical axons. This finding suggests that SARM1 deletion does not compromise the structural integrity of this specific population of adult cortical axons under normal conditions. This “morphologically silent” phenotype reflects earlier observations Thy1-YFP/Sarm1−/− mice, which similarly reported unchanged axon caliber, branching, and myelination before injury (Henninger et al., 2016; Marion et al., 2019) and is consistent with *in vitro* data showing only a context-dependent branching effect (Ketschek et al., 2022).

In terms of the response to an acute laser mediated axonal lesion, our findings align with our previous work (Canty et al., 2013a,b), indicating that laser-mediated axotomy results in maintenance of the surviving axon, and increase in synaptic TOR in the post-lesion period.

Our work in this current study examined a sub-population of axons bearing a mixture of both EPBs and TBs, with the proportion of TBs varying between axons. TBs have been described as having higher turnover rates and greater vulnerability to injury (Canty et al., 2013a; Fulopova et al., 2025). Interestingly, while our results indicate a strong positive effect of TB% on density, there was no significant correlation between TB% and synaptic plasticity.

In the absence of SARM1, baseline pre-axotomy synaptic density is comparable to wildtype axons (0.10 boutons per μm) and synapses remodel in a similar way to wildtype axons. This result mirrors several “quiet brain” datasets in the literature where constitutive SARM1 deletion left resting synapse numbers unchanged in cortex or striatum. It contrasts with the hippocampal CA1 study of Lin et al. which reported a modest increase in spine density in SARM1KO mice only after the pruning phase of adolescence (Lin et al., 2014). This indicates that SARM1 is not essential for maintaining the steady-state synaptic dynamics outside specific developmental periods and also implies the existence of effective homeostatic mechanisms, independent of SARM1, that regulate synaptic output and plasticity within a particular range, in both the baselines and post-axotomy conditions.

### 4.2 Axotomy increases synaptic turnover and loss in the surviving axon

When the axons were severed by laser lesion, we observed an increase in turnover in the proximal axon after 48 h. This increase in TOR was driven by an increase in losses, rather than gains, which remained unaltered. TOR remained elevated at our last timepoint, 6 days post-axotomy, indicating a sustained rise in synaptic remodelling, driven by losses. Increased synaptic losses after injury would need to be of a larger magnitude to be detected as a simultaneous decrease in synaptic density or be maintained over an extended period where the increased loss is only small. In the case of the data presented in this manuscript, the increased losses were detected towards the end of the experimental time window (+6 days). Extending the experimental time frame beyond 6 days post-injury would inform the duration of synaptic losses. We hypothesize that this could be interpreted in relation to the degeneration of the distal axon. The severed portion of the axon typically degenerates in a Wallerian-like pattern, fragmentating over a period of 24 h, with debris removal almost complete after 48 h. With the resulting loss of connectivity, there will be changes to local circuitry, which could impact the remaining outputs in the surviving proximal segment of the severed axon. Local circuitry synaptic remodelling might cause the pruning of some synaptic outputs in the surviving axon (increased losses) without necessarily driving an increase in gains, at least in the short term. Regardless of the mechanisms driving the increase in losses but not gains, neither of these processes were altered by the absence of SARM1 signalling in the post-lesion axon.

In previous work where we stimulated the brain with transcranial magnetic stimulation (TMS) coupled with *in vivo* imaging, similar excitatory axons in the somatosensory cortex of Thy1-GFM-M mice underwent a significant increase in turnover of boutons following a single round of TMS stimulation, with a large effect size of approximately 100%—turnover doubled within 48 h (Fulopova et al., 2025). In those studies, synaptic changes were detected using a similar volume of synaptic data—8 wildtype mice, 25 axon segments, 757 boutons, combined length 5.6 mm, compared to the current study *n* = 12 wildtype mice, 21 axons, 694 boutons, combined axonal length 8.2 mm. In the context of SARM1 signalling, Sarm1 knockdown in cultured embryonic hippocampal neurons resulted in a 20% increase in spine density, and glutamate stimulation in the presence of a Fura-2 calcium indicator, showed an increase in the calcium signal, suggesting that Sarm1 knockdown increased calcium influx via the NMDAR by altering the synaptic composition or by altering downstream signalling pathways (Lin et al., 2014). In the current study, we have detected a significant time-related effect on turnover ratio (partial-eta-squared = 0.15), however the observed effect of genotype was too small to be statistically significant and of limited biological relevance. Several *in vivo* studies examining global brain or spinal injury concur with our finding that bouton density remains unchanged by SARM1 deletion during the first week post-axotomy. In a mouse model of TBI, corpus callosum axons were preserved in SARM1 knockouts, and there was no increase cortical synaptic number within 7 days, even though distal white-matter degeneration was almost completely blocked by the removal of SARM1 signalling (Bradshaw et al., 2021). Similarly, Scheff et al. reported that synaptic loss after a TBI peaks early and recovers within a month, a pattern that WT-GFP and SARM1KO-GFP axons replicate in our study, as evidenced by their flat density curves (Scheff et al., 2005). Together with our data, these studies suggest that synapses in the proximal segment are protected against the catastrophic degeneration process that SARM1 regulates in the distal, disconnected segments.

This aligns with research showing that SARM1’s enzymatic NADase activity becomes significant during periods of metabolic or injury-induced stress. For example, in a TDP-43 ALS neurodegeneration model, mice with intact SARM1 signalling exhibited substantial progressive loss of dendritic spines within cortical neurons, whereas SARM1 deletion resulted in maintenance of dendritic spines (White et al., 2019). Similar synaptic protection after traumatic brain injury in the same mouse model has also been reported (Bradshaw et al., 2021). Recent mechanistic reviews of SARM1 signalling now describe SARM1 as a metabolic checkpoint, remaining inactive during regular remodelling but ready to initiate axon degeneration when NAD+ stress exceeds a threshold (Figley et al., 2021; Karnik and Joshi, 2025).

In the proximal surviving stump of the lesioned axons in this study, axonal transport recovered within 24–48 h, with varicosities resolving and no significant stump retraction, consistent with rapid axonal stabilisation after the initial laser-induced injury. Together, these observations support the view that the energetic/NAD+ balance in the surviving stump is restored quickly, and its perturbation is likely insufficient in duration or severity to activate SARM1 in surviving axons 48 h after injury.

### 4.3 Overall conclusions

Baseline axonal morphology and synaptic density in WT-GFP and SARM1KO-GFP cortical axons were indistinguishable, indicating that SARM1 deletion does not disrupt structural integrity or steady-state synaptic dynamics under normal conditions. Following laser-induced axotomy, synaptic turnover increased due to elevated losses in the surviving axon, yet this response—and subsequent synaptic remodelling—was unaffected by SARM1 deletion, suggesting that any synaptic effects of SARM1 deletion are either subtle, transient or non-existent.

### 4.4 Caveats

Several limitations of our study should be acknowledged. Firstly, this study was underpowered for detecting subtle synaptic phenotypes. The axotomy time course experiments are both technically demanding and time-consuming and axon numbers were therefore limited. Secondly, our last timepoint for imaging was 6 days post-axotomy. This allowed for 3 separate post-injury intervals to assess synaptic changes and may have missed any rapid changes that then resolved, or any longer-term changes. Similarly, we did not assess the time course of SARM1 activation in wildtype mice. However, there is clear evidence from other studies, that SARM1 signalling is rapidly induced after axonal injury (for example; Ko et al., 2021) and in studies using the same mice, clear attenuation of axonal pathology is described as early as 2 h after a closed head weight drop injury, peaking at 48 h post injury (Henninger et al., 2016). Finally, the experimental axons in this study were located superficially in the primary somatosensory cortex (S1), which processes tactile and sensorimotor information, with some axons likely crossing into the primary motor cortex (M1). It is well established that bouton density and synaptic dynamics can vary markedly across cortical areas, indicating differences in functional organisation and plasticity demands. For example, EPB-rich axons in the prefrontal cortex (PFC) exhibit higher synaptic density and may potentially undergo greater plasticity than those in the M1/SS1 cortex in 12-month-old mice (Fulopova et al., 2022).

## Data Availability

The original contributions presented in the study are publicly available. This data can be found here: https://doi.org/10.5281/zenodo.18344976.
